# Effect of altering the regime of oral rifampicin therapy in the treatment of persistent central serous chorioretinopathy

**DOI:** 10.12669/pjms.35.6.990

**Published:** 2019

**Authors:** Hina Loya, Hunain Ghoghari, Syed Fawad Rizvi, Abdullah Khan

**Affiliations:** 1 Dr. Hina Loya, MBBS. Layton Rahamatullah Benevolent Trust (LRBT), Free Base Eye Hospital, Korangi 2 ½, Karachi, Pakistan; 2 Dr. Hunain Ghoghari, MBBS, MRCSEd (Ophth). Layton Rahamatullah Benevolent Trust (LRBT), Free Base Eye Hospital, Korangi 2 ½, Karachi, Pakistan; 3 Prof. Syed Fawad Rizvi, MCPS (Ophth), FCPS (Ophth). Layton Rahamatullah Benevolent Trust (LRBT), Free Base Eye Hospital, Korangi 2 ½, Karachi, Pakistan; 4 Dr. Abdullah Khan, MCPS (Ophth), FCPS (Ophth). Layton Rahamatullah Benevolent Trust (LRBT), Free Base Eye Hospital, Korangi 2 ½, Karachi, Pakistan

**Keywords:** Central macular thickness (CMT), Chronic central serous chorioretinopathy (CSCR), Optical coherence tomography (OCT), Rifampicin, Visual acuity (VA)

## Abstract

**Objective:**

To study the effect of reducing the duration of rifampicin therapy in the treatment of Chronic Central Serous Chorioretinopathy.

**Methods:**

This is interventional study conducted in Layton Rahmatullah Benevolent Trust, Free Base Eye Hospital Korangi, Karachi from February 2017 - December 2018. This randomized controlled comparative study included two groups, Groups-A comprised of 48 eyes of 40 cases with Chronic Central Serous Chorioretinopathy who were given reduced dose of oral rifampicin i.e. 600mg for one month, and Group-B consisted of 43 eyes of 40 controls with Chronic Central Serous Chorioretinopathy who were given reduced dose of oral rifampicin i.e. 300mg once daily for three months as previously stated in literature. To access the effect of therapy in both the groups, pre-treatment visual acuity on the logMAR and Optical Coherent Tomography (OCT, Heidelberg spectralis) for CMT were performed and repeated on the 1^st^ and 3^rd^ month post-treatment. Patients were also followed for 6 months to access any recurrence.

**Results:**

On comparing the two groups, Group-A had improvement in VA and CMT after one month therapy of Rifampicin, Pre-treatment mean VA in Group-A was 0.85 ± 0.19 as compared to the pre-treatment mean VA in Group-B i.e. 0.74+/- 0.208, while the pre-treatment mean CMT was 609.0 ± 178.29 µm in Group-A, and 600.0 +/- 155.09 µm in Group-B respectively. After 1 month of therapy, the visual status, and CMT in Group-A was 0.29+/- 0.21 and 311.6 +/- 89.9, while Group-B, VA was 0.598 +/- 0.23 (p value 0.001%) and CMT was 512.30 +/- 148.37 (p-value 0.001%). Rifampicin was continued in Group-B till three months, and patients were re-accessed but there was no difference in VA and CMT statically. During the 3^rd^ and 6^th^ months of follow up no relapses were reported.

**Conclusion:**

This comparative study showed that the group receiving oral rifampicin 600mg for one month showed better outcome at one month and third month than the group receiving oral rifampicin at a dose of 300mg once daily for three months. This gives a better compliance and lower the risk of drug induced side effects.

## INTRODUCTION

CSCR is a common cause of decreasing vision after diabetic retinopathy, retinal vein occlusion, and age-related macular degeneration. There is no specific etiology of CSCR however corticosteroids have long been described as a precipitating factor in the epinephrine mediated vasospasm. This increases the choroidal vascular permeability leading to exudation of fluid in the sub-retinal space. Other factors which increase the endogenous steroids like Type-A personality, obstructive sleep apnea, hypertension and excessive use of oral, inhalant, topical and other exogenous corticosteroids worsens the disease.^1^,^2^ The incidence of CSCR is 10 per 100,000 cases as reported by Kitzman et al. and predominantly affect males aged 20-50 years.^3^,^4^ It presents as an acute, sub-acute or chronic visual loss. The central vision is more affected than the peripheral vision. Other symptoms include metamorphopsia, dyschromatopsia, micropsia and myopic or hypermetropic shift.^5^,^6^

Observation continues to be the first line treatment for acute CSCR because spontaneous resolution occurs in 80 to 90% of affected individuals. Withdrawal from exogenous steroids and lifestyle modifications are encouraged to decrease the endogenous steroids. Non-steroidal anti-inflammatory drugs (NSAIDs), laser photocoagulation, photodynamic therapy, intravitreal anti-VEGF therapy and newer modalities like anti corticosteroid and mineralocorticoids are other treatment options for chronic and recurrent CSCR.^7^ Recurrence is very common with 40-50% relapses in the affected eye.

Rifampicin, is an antibiotic that not only treats tuberculosis but many other diseases like leprosy, and Legionnaire’s disease when used in conjunction with other medicines. Rifampin is a bactericidal with anti-oxidative, and anti-apoptotic properties. It induces the drug metabolizing cytochrome P-450 enzyme in the liver and increases steroid metabolism thus decreasing the subretinal fluid and disease progression.^8^ Few adverse reactions associated with Rifampicin treatment include fever, gastrointestinal disturbances, rashes, immunological reaction and hepatotoxicity. Taking Rifampicin usually causes certain bodily fluids to become orange and red in color, it is a benign side effect and the patients should be counselled before starting the treatment.

Rifampicin, at high doses for three months has been known to improve Chronic CSCR, but by increasing chances of side effects. Our study aimed to see if reduced rifampicin therapy time, has similar effect or not on VA and CMT.

## METHODS

This comparative study was carried out at the Layton Rahmatulla Benevolent Trust (L.R.B.T), Free Base Eye Hospital, Karachi, from February 2017 to December 2018. The study was approved by Hospital Ethical Review Committee (LRBT/FBEH/ERC/017 dated January 15, 2017) and informed consent was taken from all the included patients. The study had two groups, Group-A encompassed 48 eyes of 40 cases aged between 20-50 years, compared to age-match control Group-B which included 43 eyes of 40 patients. The inclusion criteria were symptoms for more than 3 months and recurrent disease, visual acuity of less than 0.2 on the logMAR chart, not improving on pinhole examination, CMT >300um on SD-OCT, normal baseline serum creatinine and liver function tests. The exclusion criteria were acute CSCR, history of trauma, any intravitreal injection, laser photocoagulation or any other ocular pathology, patient taking medicines which interact with rifampicin, any systemic illness like diabetes, hepatitis, renal insufficiency, raised creatinine or abnormal liver function tests.

Group-A were started on oral rifampicin at 600mg once daily dosing for four weeks i.e. decreasing the total time period of therapy and altering the regime. While Group-B were given oral rifampicin 300mg once daily for three months as mentioned in literature. Pre-treatment VA on the logMAR and Optical Coherent Tomography (OCT, Heidelberg spectralis) for CMT were performed and repeated on the 1^st,^ 3^rd^ and 6^th^ month post-treatment. Patients were also followed to access any recurrence.

### Statistical analysis

The statistical analysis of the data was done by the software Statistical Package for Social Sciences (SPSS) version 21. Mean and the standard deviation were calculated for quantitative variables like age of patient, visual acuity, and CMT. Frequency and percentage tables were designed for gender and laterality of eye. An unpaired t-test was run to compare the pre-treatment and post-treatment VA and CMT between the groups. A p-value ≤ 0.05 was considered statistically significant.

## RESULTS

The patients were divided into two groups, Group-A which was given rifampicin 600mg for one month, while the other group i.e. Group-B was given oral rifampicin 300mg once daily for three months. A total of 48 eyes of 40 cases and 43 eyes of 40 controls with Chronic Central Serous Chorioretinopathy were included in this study. The characteristics of both the groups are summarized in Table-I. Four patients in Group-B had adverse reactions to the drug.

**Table I:** Cases and controls: Frequency distribution of various categorical variables: n=40.

Cases= 40		

Variables	Frequency	Percentage %
***Gender***		
Male	25	62.5
Female	15	37.5
***Eye***		
Right	19	42.2
Left	26	57.7
***Laterality***		
Unilateral	35	87.5
Bilateral	5	12.5

*Controls= 40*		

*Variables*	*Frequency*	*Percentage %*

***Gender***		
Male	22	55
Female	18	45
***Eye***		
Right	20	46.5
Left	23	42.4
***Laterality***		
Unilateral	37	86.0
Bilateral	6	13.9

On comparing the two groups on the basis of pre VA and pre CMT, the results were not significant, but after one month of dosing, Group-A had improvement on VA, and CMT as compared to Group-B, which was under treatment (P-value=0.001%). While, after three months, on comparing both the groups there was no difference statistically.

On the basis of these results, it was found that the efficacy of the drug was equal after three months in the two groups, but the probability of developing serious adverse effects to rifampicin were seen in Group-B in which the dose was 300mg for three months. Hence this establishes the need to modify the modern regime, and it gives upper hand over the probability of adverse reaction with long term use of the drug.

## DISCUSSION

The role of Rifampicin in CSCR was first established by Ravage and Kirk Packo et al. in 2011 when they presented a case of tuberculosis with concurrent CSCR. The patient showed remarkable visual improvement while on multi-drug anti-tuberculous therapy (ATT) but the visual status deteriorated after completion of the ATT. They reviewed and tested each ATT drugs and found that Rifampicin induces the drug metabolizing cytochrome P-450 enzyme in the liver^9^-^11^ and increases steroid metabolism consequently decreasing the subretinal fluid and disease progression which was later confirmed by the work of Steinle NC and their colleagues.^12^ Several studies have highlighted the efficacy of medication in chronic CSCR.^13^,^14^

Shulman et al. study comprised of 14 eyes in 12 patients with recurrent CSCR which were previously treated with intravitreal injections, PDT and Laser Photocoagulation. They revealed improvement in vision as well as resolution of subretinal fluid at one month post treatment in only about 9 (64%) cases, but they continued the drug for three months, therefore this improvement was maintained over a period of three months. Adverse reactions to the drug were observed in 14% and had to be discontinued.^15^ In contrast, our study did not include previously treated patients with PDT, laser or intravitreal injections. We noticed better results after one-month therapy i.e. Group-A which was statistically significant.

Khan et al. in their study of 38 eyes in 31 patients prescribed rifampicin 300mg for three months to patients primarily with recent onset CSCR and found that 74% of the patients improved in visual acuity and CMT and also no adverse effects were noted during the study.^16^ The results in the above study were higher because their 74.2% cases were acute CSCR which usually resolves in 80% of cases without any treatment^17^,^18^ but our study included only chronic CSCR and there is significant improvement even after change of regime of the drug.

A recent study by Vankatesh et al. comprised of nine eyes with chronic CSCR, and were prescribed 600mg rifampicin for three months’ period. Four eyes had visual improvement and resolution of fluid was seen in all the patients after months. There was no recurrence or adverse effects^19^ but our study showed better results with 600mg rifampicin in one month. And the number of cases were also more.

In the follow-up period of six months none of the patients had recurrence of the disease and no adverse reactions of the drug were seen in Group-A. Rifampicin in higher doses causes hepatotoxicity as shown by Nelson et al in their case report and Ramappa et al.^20^,^21^ However, we did not come across any case of hepatotoxicity in our prescribed dose of 600mg for one month during the entire six months’ period. Mildly raised LFTs were seen in Group-B after one-month therapy in four patients, but it was not statistically significant.

Optimal dosage of rifampicin in treating chronic CSCR is not known, but in our study we compared the two different regimes of Rifampicin therapy, and found that Group-A had remarkable results in one-month therapy. Our work differs from other studies because we compared two different regimes to know which is more effective and less burden on patients. We also followed the patients for 6 months and included chronic i.e. more than six months’ duration as well as recurrent CSCR.

### Limitations of the study

The limitation to our study was small sample size, and we did not include any previously treated patients.

## CONCLUSION

Our study showed convincing results in favor of rifampicin therapy for only one month duration, rather than continuing for three months. Rifampicin has side effects, and can cause drug interactions, therefore proper medical history, drug history, baseline LFTs and follow-up is mandatory. Hence, this establishes the need to modify the modern regime of the therapy.

### Authors’ Contribution:

**HL** conceived, designed, did data collection and manuscript writing.

**HG** did statistical analysis & editing of manuscript.

**SFR and AK** did review and final approval of manuscript and are responsible for integrity of research.
